# MBSCL-Net: Multi-Branch Spectral Network and Contrastive Learning for Next-Point-of-Interest Recommendation

**DOI:** 10.3390/s25185613

**Published:** 2025-09-09

**Authors:** Sucheng Wang, Jinlai Zhang, Tao Zeng

**Affiliations:** 1 Elite Engineering School, Changsha University of Science and Technology, Changsha 410114, China; 202227040123@csust.edu.cn (S.W.); zengtao@stu.csust.edu.cn (T.Z.); 2 College of Mechanical and Vehicle Engineering, Changsha University of Science and Technology, Changsha 410114, China

**Keywords:** next POI recommendation, attention mechanism, Fourier transform, contrastive learning

## Abstract

Next-point-of-interest (POI) recommendation aims to model user preferences based on historical information to predict future mobility behavior, which has significant application value in fields such as urban planning, traffic management, and optimizing business decisions. However, existing methods often overlook the differences in location, time, and category information features, fail to fully utilize information from various modalities, and lack effective solutions for addressing users’ incidental behavior. Additionally, existing methods are somewhat lacking in capturing users’ personalized preferences. To address these issues, we propose a new method called Multi-Branch Spectral Network with Contrastive Learning (MBSCL-Net) for next-POI recommendation. We use a multihead attention mechanism to separately capture the distinct features of location, time, and category information, and then fuse the captured features to effectively integrate cross-modal features, avoid feature confusion, and achieve effective modeling of multi-modal information. We propose converting the time-domain information of user check-ins into frequency-domain information through Fourier transformation, directly enhancing the low-frequency signals of users’ periodic behavior and suppressing occasional high-frequency noise, thereby greatly alleviating noise interference caused by the introduction of too much information. Additionally, we introduced contrastive learning loss to distinguish user behavior patterns and better model personalized preferences. Extensive experiments on two real-world datasets demonstrate that MBSCL-Net outperforms state-of-the-art (SOTA) methods.

## 1. Introduction

With the widespread use of GPS-enabled mobile devices and the development of location-based social networks (LBSNs), people are increasingly willing and able to share information about their activities. The large amount of check-in information accumulated as a result has led to the rapid development of next-POI recommendation systems, with many studies focusing on this task [1,2]. The development of next-POI recommendation systems provides important insights for traffic congestion management and urban planning, and can also help companies improve their advertising strategies [3].

In early studies, the focus was primarily on Markov chains (MCs), such as MMC [4], which utilized Markov chains to model the transition patterns of location sequences. With the advancement of deep learning, recurrent neural networks (RNNs) and their variants have been widely adopted in research due to their effectiveness in modeling sequence data, particularly for capturing users’ short-term preferences [5,6,7]. Later, the success of the Transformer architecture [8] also led to the application of attention mechanisms, with some studies [9,10] introducing attention mechanisms to model users’ long-term and short-term preferences. Other studies [11,12] referenced self-attention and multihead attention. Subsequently, the powerful ability of graph neural networks [13] to process spatial–structural data was utilized to model spatiotemporal relationships between locations [14], gaining widespread application [15,16,17]. Recently, some studies [18] have applied frequency-domain processing to time series. Other studies, such as POIFormer [19], SLS-REC [20], and CLSPRec [21], have introduced contrastive learning for next-POI recommendation.

These methods have been hugely successful, but when introducing various types of information such as location, time, and category to address data sparsity, they often overlook the differences in characteristics between them, fail to model each type of information in a targeted manner, do not make full use of user information, and are insufficient in modeling user interests. Additionally, while the application of attention mechanisms has driven the rapid development of next-POI recommendation systems, their large parameter scale may lead to the issue of overfitting [22]. Furthermore, the simple attention mechanism struggles to handle the inherent noise issues in time-series data [23]. There is also the challenge of better modeling users’ personalized preferences. Existing models have not effectively addressed the issues of gradient vanishing and training difficulties when handling long sequences.

To address the above issues, we propose a method called Multi-Branch Spectral Network with Contrastive Learning (MBSCL-Net) for next-POI recommendation. In MBSCL-Net, to capture users’ different preferences in terms of location, time, and POI category information, we use a multihead attention mechanism to process different types of information separately, modeling their respective fine-grained association patterns. When users have only a few check-in records, it is often difficult to capture their preference information. Additionally, to mitigate the inherent noise in time-series data, we transform the user’s check-in time-domain data into the frequency domain using the Fourier transform. In the frequency domain, we enhance the key frequency components through adaptive filtering while suppressing noise signals. This method significantly reduces noise interference and improves signal clarity. To address potential information loss during data transmission and mitigate gradient vanishing and gradient explosion issues when processing long sequences, we fuse the original data with the processed data using skip connections. Finally, we employ contrastive learning with a contrastive loss function to better capture users’ personalized preferences, enabling more accurate recommendations for the next POI.

In summary, the contributions of this paper are as follows:

- We took into account the differences in characteristics between different types of information in user check-in records. We used a multihead attention mechanism to independently process various types of information in order to capture their different characteristics.
- MBSCL-Net effectively utilizes the Fourier transform to convert check-in information from the time domain to the frequency domain.
- We use contrastive learning to better capture users’ personalized preferences and improve the accuracy of recommendations.
- Extensive experiments conducted on two real-world datasets (NYC and TKY) demonstrate the superior performance of MBSCL-Net. The results show that our model significantly outperforms existing SOTA methods.

The rest of this paper is organized as follows: First, Section 2 reviews related work. Then, Section 3 introduces the MBSCL-Net model we propose. Section 4 presents the experimental results. Finally, Section 5 summarizes this paper and outlines future work.

## 2. Related Work

### 2.1. Next-POI Recommendation

Next-POI recommendation aims to recommend the next destination for users by combining their long-term preferences based on historical behavior and the recent spatiotemporal context. The methods used in earlier studies have been widely applied in other sequential recommendation tasks, such as Markov chains and their variants [24,25]. Cheng et al. [25] proposed a personalized Markov chain model, FPMC-LR, to decompose user behavior in sequence data to obtain personalized preferences and predict the next POI in a time series. Meanwhile, Zhang et al. [26] proposed an additive Markov chain to simulate sequential influence propagation and predict sequential transmission probabilities. Other studies [27,28] utilize matrix decomposition for POI prediction. However, these early methods have limited modeling capabilities and cannot effectively capture user preferences.

In recent years, with the development of deep learning, many studies have been conducted based on RNNs and their variants due to their excellent modeling capabilities. Liu et al. [6] proposed STRNN, a pioneering work that incorporates spatial and temporal information about user visits into RNNs to predict human flow behavior. Some methods combine attention mechanisms to enhance model performance. In DeepMove [9], a multi-module embedding method is used to convert sparse features into dense representations, followed by combining the attention mechanism with RNN to capture users’ long-term and short-term preferences. Wu et al. [29] proposed the PLSPL model, which attempts to model category information and then uses the attention mechanism to learn users’ long-term preferences and LSTM to learn short-term preferences. Sun et al. [30] proposed LSTPM, which combines context-aware non-local networks and geographically expanded RNNs to model users’ long-term and short-term preferences. Luo et al. [10] proposed STAN, which combines self-attention mechanisms with spatiotemporal correlation matrices to learn non-adjacent locations and noncontinuous check-ins. PG^2^Net [31] combines attention mechanisms with Bi-LSTM to separately model users’ collective preferences and personalized preferences through spatiotemporal dependencies. Li et al. [32] proposed a multi-level collaborative neural network model, MCN4Rec, which captures complex heterogeneous relationships between different pieces of information to recommend the next location.

However, the attention mechanism used in the above methods struggles to handle the inherent noise problems in time-series data, resulting in suboptimal model performance.

### 2.2. Frequency-Aware Time-Series Forecasting

Recently, to address the issue of noise in time-series data, some studies have attempted to incorporate frequency-domain information and achieved good results. TSLA-Net [18] proposed adaptive spectral blocks and interactive convolution blocks for adaptive denoising in the frequency domain, enhancing periodic modeling capabilities. TimesNet [33] extracts periodicity through the Fourier transform (FFT) and then performs convolution operations on the periodic signal. FreTS [34] utilized the frequency domain to capture global dependencies and perform energy compression, effectively capturing time-series patterns. Additionally, FITS [35] directly trains sequences using fully connected layers in the frequency domain, achieving performance improvements while maintaining a small number of parameters.

Inspired by these works, we designed a spectrum block that converts time-domain information to the frequency domain through Fourier transform (FFT) and then filters out noise through adaptive filters.

### 2.3. Contrastive Learning

Contrastive learning has also made significant progress in time series [36,37]. Contrastive learning is an unsupervised learning method that learns data representations by maximizing the similarity between relevant samples and minimizing the similarity between irrelevant samples. Contrastive learning methods have been widely applied in computer vision, natural language processing, and time-series prediction. SimCLR [38] generates augmented views by performing data augmentation on the original data and learns through a simple and effective framework. In time series, TS2Vec [39] better captures contextual information through hierarchical contrastive learning. TSTCC [40] generates positive sample pairs from related sequences and negative sample pairs from unrelated time series, ultimately learning local and global features by maximizing the similarity of positive sample pairs.

## 3. Methodology

**Figure 1:**
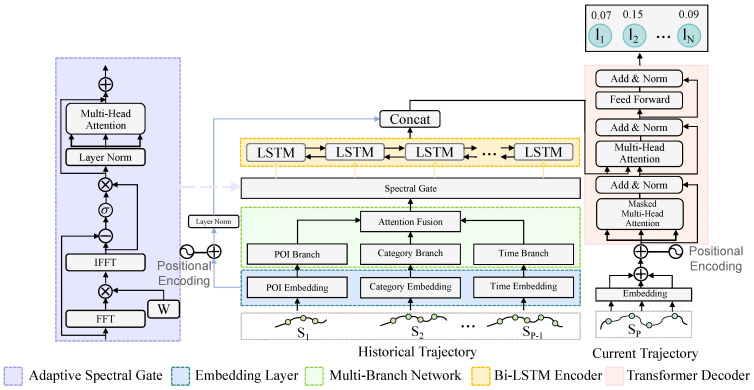
The overview of our proposed MBSCL-Net.

In this section, we provide a detailed introduction to our model. As shown in Figure 1, the MBSCL-Net mainly consists of five parts: Multi-Branch Attention Fusion Network, Adaptive Spectral Gate, LSTM encoder, Transformer decoder, skip connection, and contrastive learning module.

### 3.1. Problem Formulation

We define the user set U = {$U_{1}$, $U_{2}$, …, $U_{N}$}, the location set l = {$l_{1}$, $l_{2}$, …, $l_{L}$}, and the POI category set C = {$C_{1}$, $C_{2}$, …, $C_{C}$}. Then we define some concepts used in this paper.

**Definition** **1** (Check-in)**.** *A check-in record is a tuple q = ($U_{i}$, $l_{j}$, $C_{k}$, $t_{m}$), indicating that user $U_{i}$ visited location $l_{j}$ of category $C_{k}$ at time $t_{m}$.*

**Definition** **2** (Session)**.** *We define all check-in records generated by a user as $Q_{u}$ = ($q_{u}^{1}$, $q_{u}^{2}$, $q_{u}^{3}$, …), where $q_{u}^{i}$ represents the i-th check-in record of user U. We divide the check-in records of a user within a certain period of time into a session S, and the length of each session S may vary.*

**Definition** **3** (Next-POI Recommendation)**.** *We define next-POI recommendation as follows: given a user $U_{i}$ and its historical check-in records $Q_{u}$, the task is to recommend the top-K points of interest that the user is likely to visit at the next timestamp.*

### 3.2. Multi-Branch Attention Fusion Network

**Figure 2:**
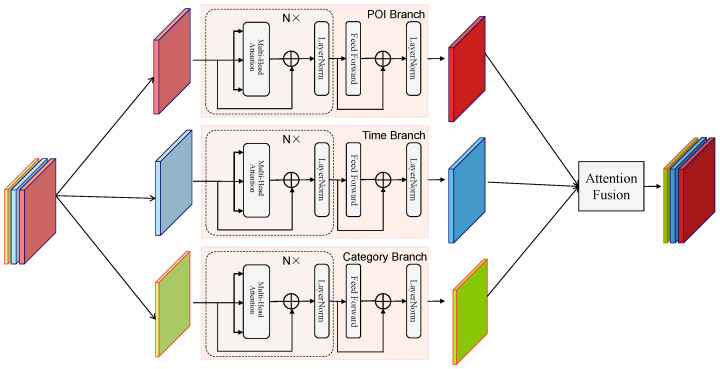
The framework of the Multi-Branch Attention Fusion Network.

The structure of the Multi-Branch Attention Fusion Network is shown in Figure 2, where *N* is set to 2. We use light-colored solid figures to represent the original embedded data, with different colors representing different embedding modalities, and then use dark-colored solid figures to represent the feature-enhanced modalities obtained after capturing features through each branch. The Multi-Branch Attention Fusion Network consists of three branches—POI Branch, Time Branch, and Category Branch—and a final Branch Fusion module. It first splits various embedded information into different branches, each focusing on a specific modality, and then fuses them together.

From human trajectory data, we can learn a great deal about human movement patterns, but due to the sparsity of trajectory sequences, we adopted the embedding method described in [9]. For user ID, location, and POI category information, based on word2vec [41], we map these sparse data into low-dimensional feature dense vectors, represented as $e_{u}\in R^{\left|N\right|\times D_{u}}$, $e_{l}\in R^{\left|L\right|\times D_{l}}$, and $e_{c}\in R^{\left|C\right|\times D_{c}}$, respectively. Here, |N| denotes the number of users, |L| denotes the number of locations, |C| denotes the number of location categories, and $D_{u}$, $D_{l}$, and $D_{c}$ represent the embedding dimensions of the corresponding features. For timestamp information, since it is continuous and cannot be directly embedded, we first divide the 24 h of a day into 24 time segments from 0 to 23 to represent weekdays, and then use 24 time segments from 24 to 47 to represent weekends, thereby distinguishing between weekdays and weekends. We then map the timestamps to the corresponding time segments and finally encode them, represented as $e_{t}\in R^{48\times D_{t}}$, with a dimension of $D_{t}$.

In previous studies, there has been a lack of further capture of embedded features, often simply concatenating various embedded information through various methods. While progress has been made at the basic feature representation level, the modal differences between multi-source heterogeneous features have been widely overlooked. Furthermore, this coarse-grained feature mixing method, which fuses embedded vectors such as geographic coordinates, timestamps, and POI categories, through simple concatenation or weighted summation, can lead to two issues: first, the semantic orthogonality of features across different dimensions is disrupted, causing temporal–spatial patterns and semantic information to become feature-confused; second, the fine-grained associative patterns unique to each dimension are difficult to model in a targeted manner. To address this issue, we designed the Multi-Branch Attention Fusion Network module. Liu et al. [42] proposed a multi-behavioral sequential recommendation model called MAINT, which makes recommendations to users by extracting different preferences from target behaviors. This model has achieved significant results, proving the feasibility of our method.

POI Branch is a component specifically designed to extract geospatial correlation features in multi-branch spatiotemporal modeling networks. This module employs a deep attention mechanism [43,44] to explicitly model the spatial dependencies between POIs in user movement trajectories, including geographic proximity, regional functionality, and potential spatial access patterns. Its design objective is to address the limitations of traditional sequence models in modeling local geographic context, providing fine-grained spatial semantic representations for personalized location recommendations.

The input to this module is the raw embedding $e_{l}$ of the POI sequence, denoted here as *X*. First, it goes through two layers of multihead attention. In each layer, multiview feature projection is performed to generate the Query, Key, and Value matrices:

(1) $$Q=XW_{Q},\;K=XW_{K},\;V=XW_{V}.$$

Among them, $W_{Q},W_{K},W_{V}\in R^{D\times D}$ are learnable parameters. Then, the attention score is obtained through the scaled dot-product attention operation, with the specific formula as follows:

(2) $$\mathrm{Attention}\left(Q,K,V\right)=\mathrm{Softmax}\left(\frac{QK^{T}}{\sqrt{D/H}}\right)V,$$

where *H* represents the number of attention heads. In addition, layer normalization and residual connections are applied within the attention module. The formula is as follows:

(3) $$X_{\mathrm{attn}}=\mathrm{LayerNorm}\left(X+\mathrm{Attention}\left(Q,K,V\right)\right).$$

When people select the next POI, they often exhibit path dependency in their movement trajectories, such as having fixed commuting routes. For example, most people have a fixed route from home to the subway station and then to the office. Given this phenomenon, we use dot-product attention operations to adaptively learn the transition probabilities between POIs, suppressing low-probability unreasonable transitions and capturing regular patterns in users’ historical visit paths. We have set up two attention layers. The first-layer attention primarily focuses on adjacent POIs in the sequence, modeling local interactions to represent neighboring relationships. The higher-layer attention receives the local features from the lower layer’s output, utilizing residual connections and feature propagation. The second layer can access a broader range of POIs, expanding the receptive field to capture regional functional features.

After multi-level attention refinement, the module further integrates multi-level spatial semantics through a feedforward network. The feedforward network extracts high-order spatial interaction information through expansion–compression dimensional operations, suppresses noise, and then adds the FFN output to the final features of the attention layer via residual connections to mitigate the vanishing gradient problem in deep networks, ensuring training stability. It then performs layer normalization again to ensure that the output feature scale is consistent with the remaining branches of the multi-branch network. The specific formula is as follows:

(4) $$X_{\mathrm{ffn}}=\mathrm{ReLU}\left(X_{\mathrm{attn}}W_{1}+b_{1}\right)W_{2}+b_{2},$$

(5) $$H=\mathrm{LayerNorm}(X_{\mathrm{attn}}+X_{\mathrm{ffn}}),$$

where $W_{1}$ and $W_{2}$ are trainable weight matrices, $b_{1}$ and $b_{2}$ are two bias parameters, and the dimension of the final output *H* is the same as that of the input *X*,$H\in R^{\left|L\right|\times D_{l}}$. For POI recommendation tasks, SpatialBranch significantly enhances the consistency of path prediction and avoids unreasonable candidate targets.

The architectural design of the Time Branch and Category Branch is largely similar to that of the POI Branch, with each branch adopting a hierarchical structure consisting of multihead attention mechanisms, feature enhancement, and hierarchical fusion. For the Time Branch, the lower-level time attention focuses on local continuity, while the higher-level attention integrates global periodicity. The Category Branch reinforces the semantic association modeling of POI categories through a hierarchical feature learning mechanism. This branch focuses on fine-grained category interaction patterns in the first-layer attention, enabling POIs with similar functional attributes to obtain higher association weights even if their physical locations are dispersed, thereby capturing users’ consumption intentions. The higher-level attention identifies regional functional combination features by expanding the receptive field. This module breaks through the limitations of pure distance through semantic association modeling, providing theoretical support for cross-regional same-category recommendations and cross-category functional combination recommendations, and suppressing redundant phenomena of excessive concentration of a single category in recommendation results.

After capturing the features of each branch, we achieve hierarchical fusion of multi-branch features through BranchAttentionFusion module with static and dynamic dual pathway design. When a new POI lacks historical data, the pure attention mechanism may fail due to noise or sparse data, and we obtain static weights to provide the base recommendation. In the static pathway, the module assigns learnable normalized weight parameters to each branch, generates probabilistic weight distributions via Softmax, and then weights and sums the output features of POI, Time, and Category Branches. To capture the complex behavioral patterns of users, we design a dynamic pathway to dynamically capture fine-grained semantic associations across branches using an attention mechanism. Based on the physical foundation of POI recommendations based on spatial location, user movement is strictly constrained by distance. Dynamic pathways use spatial branch features as the query benchmark, then project each branch feature as a key and value, concatenate them to form a global context, and then calculate the attention score through scaling dot-product attention to dynamically fuse the semantic information of each branch. Finally, the module sums the projected output of the dynamic pathway with the static weighted features through residual concatenation, and applies layer normalization to eliminate the feature scale differences to output the fused unified representation. The specific process is as follows:

(6) $${\left\{H_{i}\right\}}_{i=1}^{N}\in R^{B\times L\times D},$$

(7) $$H_{s}=\sum_{i=1}^{N}\mathrm{Softmax}{\left(\theta \right)}_{i}\cdot H_{i},$$

(8) $$H_{\mathrm{dy}}=\mathrm{Softmax}\left(\frac{\left(W_{q}H_{1}\right){\left(\mathrm{Concat}\left(W_{k}^{i}H_{i}\right)\right)}^{⊤}}{\sqrt{D}}\right)\cdot \mathrm{Concat}\left(W_{v}^{i}H_{i}\right),$$

(9) $$H_{f}=H_{\mathrm{dy}}+H_{s}.$$

In the equation, *N* denotes the number of branches (in this work N=3), where $H_{i}\in R^{B\times L\times D}$ represents the output tensor of the *i*-th branch. Here, *B* indicates the batch size, *L* stands for the length of historical behavior sequence, and *D* is the unified feature dimension. The symbol θ denotes the branch static weight parameters. The matrices $W_{q}$, $W_{k}^{i}$, and $W_{v}^{i}$ are linear transformation matrices for generating queries, keys, and values, respectively. $H_{s}$ represents the weighted sum result from the static pathway, while $H_{\mathrm{dy}}$ denotes the dynamically fused result through attention mechanisms in the dynamic pathway. $H_{f}$ constitutes the ultimate output, formed by layer-normalized integration of both static and dynamic fusion results.

### 3.3. Adaptive Spectral Gate

**Figure 3:**
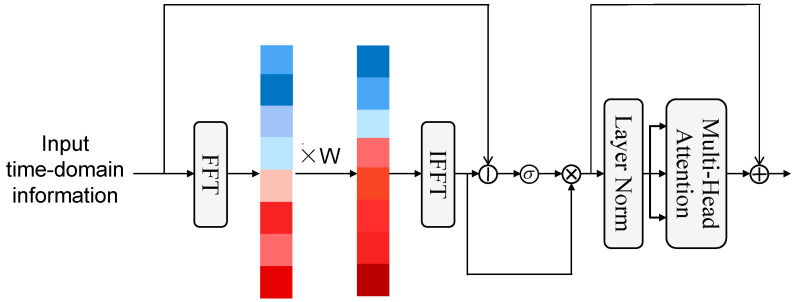
The framework of the Adaptive Spectral Gate.

Inspired by [33], we proposed an Adaptive Spectral Gate module based on frequency-domain signal processing to address the coexistence of periodic patterns and random noise in users’ mobile behavior. The specific structure of this module is shown in Figure 3. The colored blocks in the figure represent frequencies from low to high, from bottom to top. We use dark red to indicate higher weights, with the color gradually lightening to light blue and then darkening to dark blue to indicate weights from high to low. This module projects user behavior sequences into the frequency domain using fast Fourier transform (FFT) and performs global spectral modulation using learnable adaptive filters. The design also provides time–frequency dual-domain collaborative modeling capability to effectively decouple long-term behavioral patterns from short-term random fluctuations.

This is accomplished by first performing a real fast Fourier transform on the input time-domain signal along the time dimension:

(10) $$\tilde{X}=F_{r}\left(H_{f}\right).$$

The input is a time-domain user behavior sequence $H_{f}\in R^{B\times L\times D}$, where *B* is the batch size, *L* is the sequence length, and *D* is the feature dimension. Output frequency-domain complex signal $\tilde{X}\in R^{B\times K\times D}$ (K=⌊L/2⌋+1) retains the non-redundant frequency components after conjugate symmetry to reduce computational effort, and orthogonal normalization is used to ensure energy conservation. This decomposes the user behavior sequence into different frequency components, with low frequencies corresponding to long-term patterns and high frequencies corresponding to short-term fluctuations. Since high-frequency signals usually represent rapid fluctuations that deviate from the underlying trend, this can make the data more random and difficult to interpret [45]; we propose a frequency-domain adaptive filter that generates dimension-independent filters through a trainable complex weight matrix, constraining the real and imaginary parts of each dimension to the (0, 1) interval using the Sigmoid function. Define the learnable complex filter $H\in R^{D}$ whose real and imaginary parts of each dimension *d* are parameterized by the weights after Sigmoid activation, respectively:

(11) $$\begin{array}{cc} H_{d} & =\sigma \left(W_{0,d}\right)+j\sigma \left(W_{1,d}\right), \end{array}$$

where σ(·) is a Sigmoid function that learns the real and imaginary weights of the parameter matrices $W_{0,d}$ and $W_{1,d}$ corresponding to the feature dimension *d*. The weights are then applied to the frequency-domain signal element by element. Then complex multiplication is applied to the frequency-domain signal element by element:

(12) $${\tilde{X}}_{b,k,d}^{\prime }={\tilde{X}}_{b,k,d}\cdot H_{d}.$$

We expand the result into real and imaginary part weights:

(13) Re(X˜′)=Re(X˜)·σ(W0)−Im(X˜)·σ(W1)Im(X˜′)=Re(X˜)·σ(W1)+Im(X˜)·σ(W0),

(14) X˜′=Re(X˜′)+j·Im(X˜′).

Finally, we reconstruct the time-domain signals by inverse transformation and keep the length of the output sequence consistent with the input:

(15) $$X^{\prime }=F_{r}^{-1}\left({\tilde{X}}^{\prime }\right).$$

We obtain the result $X^{\prime }\in R^{B\times L\times D}$. To further optimize the feature characterization, the module further introduces a difference-driven attention gating mechanism to generate dynamic weights from the feature differences before and after spectral processing:

(16) $$\begin{array}{cc} \Delta X & =X^{\prime }-H_{f}, \end{array}$$

(17) $$\begin{array}{cc} A & =\sigma (\Delta X), \end{array}$$

(18) $$\begin{array}{cc} X_{\mathrm{freq}} & =X^{\prime }\cdot A. \end{array}$$

In the above equation, if ΔX>0, it represents that the frequency-domain enhancement is effective and amplifies the contribution of the dimension, and if ΔX<0, the frequency-domain processing is distorted and attenuates the impact of the dimension. This is followed by a dual-domain synergy with the multihead time-domain attention module:

(19) $$\begin{array}{cc} Q & =K=V=\mathrm{LayerNorm}(X_{\mathrm{freq}}), \end{array}$$

(20) $$\begin{array}{cc} X_{\mathrm{time}} & =\mathrm{Softmax}\left(\frac{QK^{⊤}}{\sqrt{D}}\right)V. \end{array}$$

The final output of the time–frequency synergy feature is obtained by residual concatenation:

(21) $$X_{\mathrm{out}}=X_{\mathrm{freq}}+X_{\mathrm{time}}.$$

The final result is $X_{\mathrm{out}}\in R^{B\times L\times D}$.

This module adapts through backpropagation by jointly optimizing the trainable parameter $H_{d}$ with other parts of the network, enabling it to adaptively amplify task-relevant global spectral patterns and suppress noise-dominated spectral components. Specifically, if high-frequency components are generally associated with noise, this mechanism automatically weakens the spectral weights of the corresponding features. Overall, this module indirectly emphasizes low-frequency long-term patterns and suppresses high-frequency short-term noise in user behavior sequences by utilizing learnable global modulation combined with data-driven frequency-domain attention and time-domain multihead attention.

### 3.4. Skip Connection

Deep learning models often face the dual challenges of feature degradation and vanishing gradients when processing long trajectory data. In our model, the main path passes through a multi-branch network and an Adaptive Spectral Gate module, which offer significant advantages in feature extraction and noise filtering. However, during propagation, degradation issues may arise, leading to the loss of certain features, and there is also the risk of vanishing gradients. The residual network proposed by He et al. [46] largely addresses this issue. Its core idea is to directly connect the input to the output in certain layers of the network, enabling information to jump and propagate between different layers. Therefore, we designed a skip connection section inspired by this method, aiming to resolve the issues of gradient vanishing and training difficulties.

In the specific implementation method, we first concatenate the original POI, time, and category of the historical sequence, and then inject the concatenated result into the sequence order information through a position-encoding module. We use sine and cosine functions of different frequencies:

(22) $$\begin{array}{cc} \mathrm{PE}(pos,2i) & =\sin\left(\frac{pos}{10000^{2i/d_{\mathrm{model}}}}\right), \end{array}$$

(23) $$\begin{array}{cc} \mathrm{PE}(pos,2i+1) & =\cos\left(\frac{pos}{10000^{2i/d_{\mathrm{model}}}}\right), \end{array}$$

where pos is the position and *i* is the dimension. This corresponds to the input of the subsequent Transformer decoder. Then concatenate it with the output result of the encoder.

This module concatenates the original shallow local features with the extracted deep global features in the channel dimension. In addition to preventing information loss and gradient vanishing during deep network processing, it also forms complementary and enhanced feature representations. Furthermore, this module accelerates the training process.

### 3.5. LSTM Encoder and Transformer Decoder

In order to better capture contextual information, unlike earlier work that directly used LSTM encoding, we use Bi-LSTM for encoding. The Bi-LSTM calculation is specifically as follows:

(24) $$\begin{array}{cc} \vec{h_{i}} & =\mathrm{LSTM}\left(E_{i},{\vec{h}}_{i-1}\right), \end{array}$$

(25) $$\begin{array}{cc} \overset{\leftarrow }{h_{i}} & =\mathrm{LSTM}\left(E_{i},{\overset{\leftarrow }{h}}_{i-1}\right), \end{array}$$

(26) $$\begin{array}{cc} h_{i} & =\left[\vec{h_{i}}⊕\overset{\leftarrow }{h_{i}}\right], \end{array}$$

where $h_{i}$ denotes the hidden information of record $q_{i}$ in the historical trajectory, and ⊕ denotes the concatenation of forward and backward output combinations. The Bi-LSTM encoder is used to capture bidirectional contextual dependencies in each user session. Bi-LSTM can naturally model local and sequential dependencies, favoring temporal order, which is particularly useful for human mobile data, where short-range patterns are critical.

The Transformer is a deep learning model architecture designed for natural language processing and other sequence-to-sequence tasks, first proposed by Vaswani et al. in 2017 [8]. The core idea of this model is the self-attention mechanism, which enables the model to consider information from different positions within an input sequence during processing. The Transformer model consists of multiple self-attention layers, which can process the input in parallel. We adopt the Transformer as the decoder, first concatenating the target POI, time, and category embeddings to obtain a combined spatiotemporal embedding representation, and then applying a position-encoding module to inject sequence order information. We use sine and cosine functions of different frequencies:

(27) $$\begin{array}{cc} \mathrm{PE}(pos,2i) & =\sin\left(\frac{pos}{10000^{2i/d_{\mathrm{model}}}}\right), \end{array}$$

(28) $$\begin{array}{cc} \mathrm{PE}(pos,2i+1) & =\cos\left(\frac{pos}{10000^{2i/d_{\mathrm{model}}}}\right), \end{array}$$

where pos is the position, and *i* is the dimension. The input is then fed into a two-layer Transformer decoder, each layer comprising three core components: a causal self-attention mechanism using a lower triangular mask matrix to ensure that position *i* can only attend to elements ≤i; an encoder–decoder cross-attention layer that queries the final encoded memory of the historical trajectory as key–value pairs; and a feedforward neural network to enhance nonlinear expression capabilities.

### 3.6. Contrastive Learning Loss

To better uncover user behavior patterns and travel preferences, we use contrastive learning loss, which is a user–POI contrastive learning component, with its core functionality being to enhance the semantic association between user preferences and location features through a contrastive learning mechanism. By maximizing the interaction information between users and the POIs they visit, it learns more discriminative representations. The core idea is that user embeddings should be similar to the embeddings of POIs they have visited, while being distinct from the embeddings of POIs they have not visited.

The loss’s input consists of user embeddings and POI embeddings. We first use two independent MLP networks to process user and location features for deep semantic transformation, followed by a normalization layer to stabilize the distribution. Then, through L2 normalization, the vectors are converted to unit vectors to ensure the rationality of similarity calculations. For POI sequence inputs, the loss selects the last visited location as the positive sample. The core contrastive learning calculates the cosine similarity matrix via dot-product operations, where the temperature coefficient finely tunes the steepness of the probability distribution. The cross-entropy loss function forces user features to align with their own visited POIs while pushing away similarities with other users’ POIs. The contrastive loss expression is as follows:

(29) $$L_{\mathrm{contrast}}=-\frac{1}{N}\sum_{i=1}^{N}\log\left(\frac{\exp\left(\mathrm{sim}(u_{i},p_{i})/\tau \right)}{\sum_{j=1}^{N}\exp\left(\mathrm{sim}(u_{i},p_{j})/\tau \right)}\right),$$

where *N* denotes the batch size, $u_{i}$ denotes the normalized final feature representation of user *i*, $p_{i}$ denotes the positive sample POI features corresponding to user *i*, $p_{j}$ denotes all POI features in the batch, the sim function is used to calculate the cosine similarity between two vectors, and τ is a temperature parameter used to control the smoothness of normalization.

### 3.7. Prediction

Here, we adopt a Transformer-based sequence-to-sequence architecture for multi-step POI prediction. At each time step, the temporal–spatial embeddings of the target sequence are received, combined with encoder memory and positional encoding, and gradually generated into future POI sequences through an autoregressive decoder under causal masking constraints. The main prediction head generates an accurate POI probability distribution, while an MLP serves as an auxiliary category prediction head as a regularization term, constraining the model to learn general features related to POI categories through KL divergence loss. The final multi-task loss function combines POI prediction loss, category regularization loss, and user–POI contrast loss. The final loss function is as follows:

(30) $$L_{\mathrm{final}}=L_{\mathrm{poi}}+L_{\mathrm{cat}}+\beta \cdot L_{\mathrm{contrast}},$$

where β is the weighting coefficient for the contrast loss $L_{contrast}$. This hyperparameter balances the training emphasis of different loss terms through a dynamic scheduling mechanism to optimize model performance.

## 4. Experiments

In this section, we evaluate the proposed MBSCL-Net model on two datasets, compare our proposed method with the SOTA next-POI recommendation model, and discuss the experimental results.

### 4.1. Datasets

We evaluated our model on publicly available Foursquare check-in datasets for NYC and TKY, which are widely used. Regarding privacy and ethical considerations, both datasets have been publicly released and widely used in previous studies. We strictly adhere to the terms of use of the original datasets and ensure that all experiments comply with ethical research standards. Each dataset includes a user ID, a POI ID, a category ID, a timestamp, and the latitude and longitude of the POI, among other details. In our experiments, we preprocessed the datasets by excluding POIs with fewer than 10 visits in the NYC and TKY datasets. Additionally, for each dataset, each user’s check-in records were divided into multiple sessions based on a 24 h time window. Each session must contain at least three check-ins, and users with fewer than five sessions were filtered out. Finally, we strictly followed the time sequence and used the first 80% of each user session as the training set and the remaining 20% as the test set. The statistical information of the preprocessed dataset is summarized in Table 1.

### 4.2. Baseline Models

To demonstrate the effectiveness of MBSCL-Net, we selected several mainstream next-POI recommendation models for comparisons. The baseline models are described as follows:

LSTM: LSTM is a neural network-based model, a variant of recurrent neural networks, capable of efficiently processing sequence data.

STAN [10]: STAN models spatiotemporal correlations between non-adjacent locations and non-adjacent visits using a self-attention network.

DeepMove [9]: DeepMove uses an attention mechanism to learn users’ long-term and short-term preferences by leveraging their historical and current sessions.

PLSPL [29]: A neural network model that considers category information during network construction to learn each user’s specific preferences.

LSTPM [30]: This is a location prediction model that uses a context-aware non-local network structure and a geographically extended RNN to capture users’ long-term and short-term preferences, respectively.

PG^2^Net [31]: PG^2^Net uses a bidirectional long short-term memory network based on spatiotemporal attention to learn user group and personalized preferences.

HUE-SCL [47]: HUE-SCL constructs a hypergraph based on user–interest point interaction information and utilizes the complex higher-order information embedded in the hypergraph to further embed users and deeply mine their personalized preferences.

**Figure 4:**
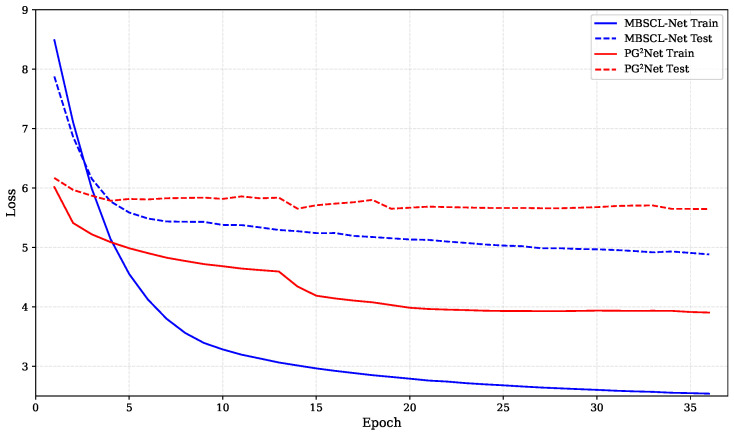
Loss comparison between MBSCL-Net and PG^2^Net during training and testing on the TKY dataset.

**Figure 5:**
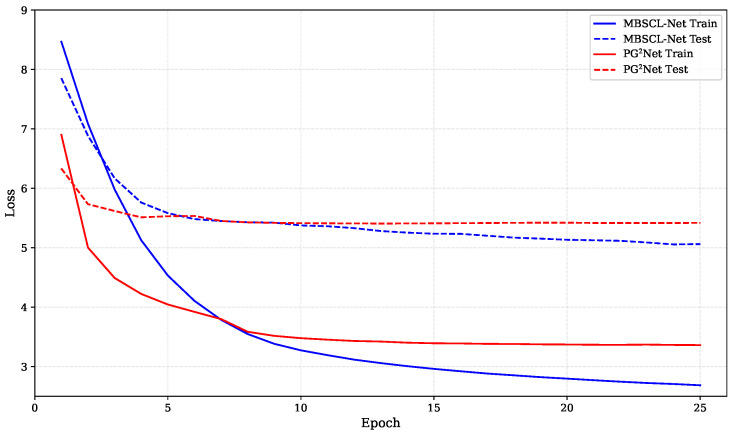
Loss comparison between MBSCL-Net and PG^2^Net during training and testing on the NYC dataset.

For our method, the embedding dimensions $D_{u}$, $D_{l}$, $D_{c}$, and $D_{t}$ for users, locations, categories, and timestamps are all equal and set to D=64. The batch size is set to 32. We use the AdamW optimizer, a gradient descent optimization algorithm, and set the initial learning rate and regularization weight to 0.001 and 1 × 10^−5^, respectively. The hidden layer dimension is set to 128, and the dropout rate is 0.3. The temperature parameter τ is set to 0.1. In Figure 4, we show the loss comparison between our model MBSCL-Net and PG^2^Net trained for the same number of epochs on the TKY dataset. In Figure 5, we show the loss comparison between our model MBSCL-Net and PG^2^Net trained for the same number of epochs on the NYC dataset. The loss curves for the training set and validation set in Figure 4 and Figure 5 remain highly consistent, indicating that there is no overfitting issue.

### 4.3. Evaluation Metrics

To compare our model with the baseline model, we adopted two commonly used evaluation metrics from previous studies [48,49]: Recall@K and NDCG@K. Recall@K is used to measure whether the correct location appears among the top-K recommended POIs, thereby assessing the accuracy of the model’s predictions. NDCG@K, on the other hand, is used to evaluate the ranking quality of the top-K recommended POIs. Higher metrics indicate better performance. For a comprehensive evaluation, we selected K = 1, 5, and 10. The formulas for Recall@K and NDCG@K are as follows:

(31) $$\begin{array}{cc} \mathrm{Recall}@k & =\frac{1}{N}\sum_{i=1}^{N}\frac{|S_{i}^{k}\cap S_{i}^{\mathrm{test}}|}{|S_{i}^{\mathrm{test}}|}, \end{array}$$

(32) $$\begin{array}{cc} \mathrm{NDCG}@k & =\frac{1}{N}\sum_{i=1}^{N}\frac{1}{z_{i}}\sum_{j=1}^{k}\frac{2^{I(S_{i}^{j}\cap S_{i}^{\mathrm{test}})}-1}{\log_{2}\left(j+1\right)}, \end{array}$$

where $S_{i}^{k}$ denotes the top-K positions recommended for user *i*, *N* denotes the number of users, $S_{i}^{\mathrm{test}}$ denotes the list of positions accessed in the test set, I(·) is the index function, $S_{i}^{j}$ denotes the *j*th position recommended in $S_{i}^{k}$, and $z_{i}$ is the maximum value in DCG@K, which is a normalization constant representing the number of prediction records for each user. For each test instance, the model ranks the ground-truth POI among all possible POIs in the dataset.

### 4.4. Main Results

The experimental results comparing the performance of our model with the baseline models on the two datasets are shown in Table 2. The best results for each metric are indicated in bold. The results show that our method outperforms all baseline models on all metrics on both datasets. In particular, our model demonstrates outstanding performance on the TKY dataset. Specifically, compared to the baseline model PG^2^Net, on the NYC dataset, Recall@1 improved by 1.78%, Recall@5 improved by 3.48%, Recall@10 improved by 4.33%, NDCG@1 improved by 1.78%, NDCG@5 improved by 2.7%, and NDCG@10 improved by 2.97%. On the TKY dataset, Recall@1 improved by 5.02%, Recall@5 improved by 14.69%, Recall@10 improved by 16.81%, NDCG@1 improved by 5.02%, NDCG@5 improved by 10.07%, and NDCG@10 improved by 10.77%. These results demonstrate the superiority and effectiveness of our model in the point-of-interest recommendation task.

LSTM is a variant of RNN that effectively alleviates the gradient vanishing problem when processing long sequence data and has stronger modeling capabilities for long-term and short-term preferences. However, the model is too simple to capture more complex user behavior patterns.

Although STAN models discontinuous check-in behavior by introducing self-attention networks, it lacks the ability to capture features across modalities and does not model personalized preferences. STAN models discontinuous check-in behavior using a special spatiotemporal embedding method, but it lacks the ability to capture continuous check-in behavior. In contrast, our model models personalized preferences through contrastive learning and captures the features of each model more precisely through a multi-branch network, resulting in superior performance.

DeepMove introduces an attention mechanism to process trajectory sequences, enhancing its ability to model user preferences. PLSPL introduces an attention mechanism and category information to address data sparsity to a certain extent. However, their modeling capabilities remain limited. Specifically, they do not consider the association information between users and POIs, and the method of directly concatenating category information introduces noise. In contrast, our method captures the association information between users and POIs through contrastive learning, thereby enhancing the modeling of user-personalized preferences. The independent processing of each modality reduces the increase in noise caused by introducing more information. Additionally, the spectral module directly identifies noise information in the frequency domain, thereby reducing interference.

LSTPM uses LSTM to model users’ long-term preferences and geographically extended LSTM to model short-term preferences. Compared to other baseline models, PG^2^Net performs well. It models user preferences using a Bi-LSTM combined with spatiotemporal attention, demonstrating stronger information capture capabilities. HUE-SCL models users as hyperedges and models the POIs they visit as nodes in these hyperedges, constructing a hypergraph to capture the interaction between users and POIs. While these models have further enhanced their modeling capabilities, they still do not account for the feature differences between different modalities, have limitations in modeling user periodic behavior, and overlook user–POI interactions. Our model captures the features of different modalities. Additionally, in terms of effectiveness, our spectral module has stronger modeling capabilities for users’ periodic behaviors and strengthens the connection between users and POIs through contrastive learning, which is why our model performs more effectively compared to others.

Our model MBSCL-Net and the baseline model PG^2^Net are compared in terms of the time required for each training round on the TKY dataset under the same experimental conditions, as shown in Table 3. The results indicate that our model requires less time for each training round and is more efficient than the baseline model PG^2^Net.

Our model enables independent processing of different modal features. When one type of feature is sparse, information from other categories can be used to supplement it, thereby alleviating the problem of data sparsity to a certain extent and reducing the introduction of noise information. We also use contrastive learning to model users’ personalized preferences. Even if a user has limited check-in data, we can still recommend points of interest (POIs) based on other users with similar preferences. Our model demonstrates superior performance when handling sparse data.

### 4.5. Ablation Study

To validate the effectiveness of different modules in the model, fifteen degraded models were obtained by decomposing MBSCL-Net. We use MB to represent Multi-Branch Attention Fusion Network, ASG to represent Adaptive Spectral Gate, CL to represent Contrastive Learning, and Skip to represent Skip Connection. The results of the ablation experiments are shown in Table 4 and Table 5.

The results show that the complete MBSCL-Net model has the best performance on both datasets, and each module plays its own role in further improving the model’s performance. Through observation, we found that Multi-Branch Attention Fusion Network and Skip Connection have a greater impact on model performance, while Contrastive Learning has a smaller impact. Based on this analysis, the following can be derived:

The significant impact of the Multi-Branch Attention Fusion Network on performance indicates that users indeed have different preferences across various modal information. Independently processing preferences for different modalities can better capture user preferences. The significant impact of Skip Connection on model performance indicates that while our model can mitigate noise interference and better model preferences through the Multi-Branch Network and Adaptive Spectral Gate modules, it also loses some original features, thereby reducing the model’s effectiveness to some extent. Contrastive Learning can better capture user–POI interactions and model users’ personalized preferences, but its effectiveness is relatively secondary compared to other modules.

We also conducted ablation experiments on the static and dynamic pathways in the Multi-Branch Attention Fusion Network module, with the results shown in Table 6.

As shown in Table 6, both the static and dynamic pathways in the Multi-Branch Attention Fusion Network module can enhance the performance of the model and are effective. Furthermore, when used in combination, they demonstrate even stronger performance.

We also verified the performance of the Bi-LSTM encoder and Transformer decoder separately by replacing the Bi-LSTM encoder with an LSTM encoder and removing the Transformer decoder. The results are shown in Table 7.

The results show that the use of both the Bi-LSTM encoder and the Transformer decoder enhances the model’s performance. Among these, the Transformer decoder plays a key role in our model.

In addition, we found that our model performed significantly better on the TKY dataset than on the NYC dataset. This may be due to the increased complexity of the model, while the NYC dataset is relatively smaller than the TKY dataset, resulting in a decline in performance.

### 4.6. Hyperparameter Analysis

In this section, we further analyze the impact of two hyperparameters—the dimension dim of the embedded feature vector and the number of attention layers in the Multi-Branch Attention Fusion Network module—on the performance of the MBSCL-Net model.

**Figure 6:**
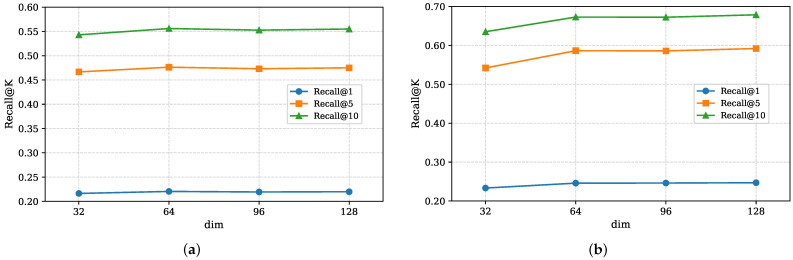
Impact of hyperparameter dim. (**a**) Impact on the NYC dataset; (**b**) impact on the TKY dataset.

The experimental results for embedding dimension dim are shown in Figure 6. In the experiment, we set the values of dim to 32, 64, 96, and 128. It can be observed that the performance is worst when dim is set to 32, possibly because the smaller embedding dimension limits the model’s learning capacity. As dim increases, the model performance gradually improves and stabilizes. When dim is set to 64, the model already achieves good performance. Further increasing the value of dim adds additional computational overhead but only results in a slight improvement in performance. Therefore, we set dim to 64.

**Figure 7:**
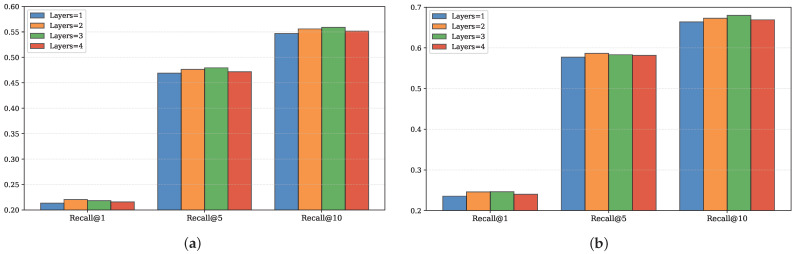
The impact of attention layers; (**a**) impact on the NYC dataset, (**b**) Impact on the TKY dataset.

The experimental results for the number of attention layers are shown in Figure 7. It can be observed that the model performance is optimal when Layers is set to 2. When the number of layers is 1, the receptive field is limited, and only basic associations between information can be established, making it impossible to capture more complex semantic relationships. This leads to underfitting of the model, requiring more layers for deeper understanding. However, when the number of layers is too high, noise may be learned, leading to overfitting of the model.

## 5. Conclusions

In this paper, we propose the MBSCL-Net model. This model uses a Multi-Branch Attention Fusion Network to independently process different modal information, capturing the features of each modality. It is also the first model to remove noise by transforming time-domain information into frequency-domain information and capture user periodic features for next-POI recommendation. Additionally, to prevent information loss during feature capture, we use skip connections to fuse the original information. Our model also enhances the modeling capability of user-personalized preferences through contrastive learning. Through experiments on two real-world datasets, we demonstrate that our model significantly outperforms current SOTA models and validate the effectiveness of its various components. In future work, we will introduce graph neural networks to handle the complex dependencies among different features, further improving the performance of next-POI recommendation.

## Figures and Tables

**Table 1:** Statistics of datasets.

Dataset	Users	POIs	Categories	Sessions
NYC	935	13,962	376	18,203
TKY	2108	21,395	354	50,306

**Table 2:** Performance comparison on NYC and TKY datasets. The values in parentheses are the standard deviations of the model.

Dataset	Model	Recall@1	Recall@5	Recall@10	NDCG@1	NDCG@5	NDCG@10
NYC	LSTM	0.1433 (0.001080)	0.2937 (0.001813)	0.3316 (0.002121)	0.1433 (0.001080)	0.2250 (0.001257)	0.2373 (0.001294)
	STAN [10]	0.1585 (0.005454)	0.3488 (0.002439)	0.3813 (0.002439)	0.1885 (0.005454)	0.2644 (0.001515)	0.2745 (0.001359)
	DeepMove [9]	0.1762 (0.001617)	0.3964 (0.001274)	0.4700 (0.001809)	0.1762 (0.001617)	0.2919 (0.001478)	0.3158 (0.001603)
	PLSPL [29]	0.1533 (0.002539)	0.3329 (0.000974)	0.3984 (0.001574)	0.1533 (0.002539)	0.2486 (0.001238)	0.2699 (0.001128)
	LSTPM [30]	0.1823 (0.000711)	0.4301 (0.000585)	0.5228 (0.001286)	0.1823 (0.000711)	0.3126 (0.000319)	0.3428 (0.000345)
	PG^2^Net [31]	0.2027 (0.000840)	0.4415 (0.003950)	0.5126 (0.006228)	0.2027 (0.000840)	0.3303 (0.001775)	0.3535 (0.002393)
	HUE-SCL [47]	0.2075	0.4816	0.5489	0.2075	0.3461	0.3667
	MBSCL-Net	**0.2205 (0.000973)**	**0.4763 (0.001524)**	**0.5559 (0.001838)**	**0.2205 (0.000973)**	**0.3573 (0.001268)**	**0.3832 (0.001458)**
TKY	LSTM	0.1554 (0.001384)	0.3119 (0.000840)	0.3569 (0.011100)	0.1554 (0.001384)	0.2402 (0.000775)	0.2537 (0.000616)
	STAN [10]	0.1616 (0.006388)	0.2384 (0.011248)	0.2636 (0.008391)	0.1616 (0.006388)	0.2010 (0.004606)	0.2093 (0.004168)
	DeepMove [9]	0.1637 (0.000480)	0.3582 (0.000935)	0.4244 (0.001018)	0.1637 (0.000480)	0.2673 (0.000558)	0.2888 (0.000579)
	PLSPL [29]	0.1670 (0.001506)	0.3517 (0.001239)	0.4299 (0.001175)	0.1670 (0.001506)	0.2642 (0.000758)	0.2896 (0.000850)
	LSTPM [30]	0.1752 (0.000436)	0.3922 (0.000916)	0.4726 (0.001687)	0.1752 (0.000436)	0.2901 (0.000661)	0.3162 (0.000932)
	PG^2^Net [31]	0.1957 (0.001220)	0.4395 (0.000910)	0.5048 (0.001383)	0.1957 (0.001220)	0.3266 (0.000892)	0.3479 (0.001056)
	HUE-SCL [47]	0.2135	0.4597	0.5283	0.2135	0.3391	0.3601
	MBSCL-Net	**0.2459 (0.000892)**	**0.5864 (0.001364)**	**0.6729 (0.001535)**	**0.2459 (0.000892)**	**0.4273 (0.001071)**	**0.4556 (0.001228)**

**Table 3:** Comparison of the time required for each training round on the TKY dataset between MBSCL-Net and PG^2^Net under the same experimental conditions.

Model	PG^2^Net	MBSCL-Net
Training Time (s)	3754	1886

**Table 4:** Ablation results on NYC.

MB	ASG	CL	Skip	Recall@1	Recall@5	Recall@10	NDCG@1	NDCG@5	NDCG@10
				0.1848	0.4010	0.4752	0.1848	0.3003	0.3245
✔				0.1988	0.4476	0.5246	0.1988	0.3346	0.3597
	✔			0.1939	0.4208	0.5009	0.1939	0.3131	0.3392
		✔		0.1937	0.4298	0.5079	0.1937	0.3192	0.3447
			✔	0.1967	0.4430	0.5249	0.1967	0.3275	0.3541
✔	✔			0.2021	0.4419	0.5187	0.2021	0.3300	0.3550
✔		✔		0.2026	0.4457	0.5180	0.2026	0.3322	0.3558
✔			✔	0.2061	0.4567	0.5399	0.2061	0.3395	0.3666
	✔		✔	0.1997	0.4478	0.5329	0.1997	0.3312	0.3590
	✔	✔		0.1967	0.4214	0.5063	0.1967	0.3163	0.3406
		✔	✔	0.1992	0.4474	0.5289	0.1992	0.3310	0.3575
✔	✔	✔		0.2037	0.4369	0.5128	0.2037	0.3282	0.3529
✔	✔		✔	0.2079	0.4664	0.5473	0.2079	0.3455	0.3719
✔		✔	✔	0.2144	0.4733	0.5567	0.2144	0.3522	0.3794
	✔	✔	✔	0.2014	0.4516	0.5348	0.2014	0.3344	0.3615
✔	✔	✔	✔	**0.2205**	**0.4763**	**0.5559**	**0.2205**	**0.3573**	**0.3832**

**Table 5:** Ablation results on TKY.

MB	ASG	CL	Skip	Recall@1	Recall@5	Recall@10	NDCG@1	NDCG@5	NDCG@10
				0.2141	0.4912	0.5768	0.2141	0.3611	0.3890
✔				0.2261	0.5321	0.6193	0.2261	0.3890	0.4174
	✔			0.2296	0.5491	0.6390	0.2296	0.3993	0.4286
		✔		0.2259	0.5280	0.6143	0.2259	0.3870	0.4151
			✔	0.2310	0.5428	0.6305	0.2310	0.3972	0.4258
✔	✔			0.2278	0.5290	0.6159	0.2278	0.3883	0.4166
✔		✔		0.2285	0.5398	0.6251	0.2285	0.3945	0.4222
✔			✔	0.2371	0.5693	0.6552	0.2371	0.4142	0.4422
	✔		✔	0.2361	0.5679	0.6558	0.2361	0.4128	0.4416
	✔	✔		0.2304	0.5429	0.6334	0.2304	0.4066	0.4329
		✔	✔	0.2353	0.5688	0.6567	0.2353	0.4129	0.4416
✔	✔	✔		0.2351	0.5506	0.6326	0.2351	0.4034	0.4302
✔	✔		✔	0.2416	0.5821	0.6717	0.2416	0.4228	0.4521
✔		✔	✔	0.2383	0.5731	0.6608	0.2383	0.4167	0.4455
	✔	✔	✔	0.2392	0.5783	0.6688	0.2392	0.4194	0.4490
✔	✔	✔	✔	**0.2459**	**0.5864**	**0.6729**	**0.2459**	**0.4273**	**0.4556**

**Table 6:** Static and dynamic pathway ablation experiments in the Multi-Branch Attention Fusion Network module.

Dataset	Model	Recall@1	Recall@5	Recall@10	NDCG@1	NDCG@5	NDCG@10
NYC	MB-static-only	0.2136	0.4716	0.5558	0.2136	0.3508	0.3782
	MB-dynamic-only	0.2072	0.4623	0.5464	0.2072	0.3428	0.3702
	MBSCL-Net	**0.2205**	**0.4763**	**0.5559**	**0.2205**	**0.3573**	**0.3832**
TKY	MB-static-only	0.2440	0.6043	0.6908	0.2440	0.4356	0.4639
	MB-dynamic-only	0.2431	0.6015	0.6878	0.2431	0.4342	0.4625
	MBSCL-Net	**0.2459**	**0.5864**	**0.6729**	**0.2459**	**0.4273**	**0.4556**

**Table 7:** Effect of encoder and decoder architectures on MBSCL-Net performance.

Dataset	Model	Recall@1	Recall@5	Recall@10	NDCG@1	NDCG@5	NDCG@10
NYC	MBSCL-Net (LSTM Enc)	0.2121	0.4753	0.5579	0.2121	0.3524	0.3793
	MBSCL-Net (w/o Trans Dec)	0.1722	0.3980	0.4799	0.1722	0.2916	0.3183
	MBSCL-Net	**0.2205**	**0.4763**	**0.5559**	**0.2205**	**0.3573**	**0.3832**
TKY	MBSCL-Net (LSTM Enc)	0.2408	0.5980	0.6853	0.2408	0.4308	0.4594
	MBSCL-Net (w/o Trans Dec)	0.2055	0.4541	0.5363	0.2055	0.3376	0.3644
	MBSCL-Net	**0.2459**	**0.5864**	**0.6729**	**0.2459**	**0.4273**	**0.4556**

## Data Availability

The original contributions presented in this study are included in the article. Further inquiries can be directed to the corresponding author.
